# Rabbit Model of Human Gliomas: Implications for Intra-Arterial Drug Delivery

**DOI:** 10.1371/journal.pone.0169656

**Published:** 2017-01-19

**Authors:** Huamin Qin, Miroslaw Janowski, Monica S. Pearl, Izabela Malysz-Cymborska, Shen Li, Charles G. Eberhart, Piotr Walczak

**Affiliations:** 1 Russell H. Morgan Department. of Radiology and Radiological Science, Division of MR Research, Cellular Imaging Section, The Johns Hopkins University School of Medicine, Baltimore, MD, United States of America; 2 Institute for Cell Engineering, The Johns Hopkins University School of Medicine, Baltimore, MD, United States of America; 3 Department of Pathology, Second Affiliated Hospital of Dalian Medical University, Dalian, Liaoning, China; 4 NeuroRepair Department, Mossakowski Medical Research Centre, Polish Academy of Sciences, Warsaw, Poland; 5 Division of Interventional Neuroradiology, The Johns Hopkins University School of Medicine, Baltimore, MD, United States of America; 6 Department of Neurology and Neurosurgery, Faculty of Medical Sciences, University of Warmia and Mazury, Olsztyn, Poland; 7 Department of Pathology, The Johns Hopkins University School of Medicine, Baltimore, MD, United States of America; Swedish Neuroscience Institute, UNITED STATES

## Abstract

The prognosis for malignant brain tumors remains poor despite a combination of surgery, radiotherapy, and chemotherapy. This is partly due to the blood-brain barrier, a major obstacle that prevents therapeutic agents from effectively reaching the tumor. We have recently developed a method for precise and predictable opening of the blood-brain barrier via the intra-arterial administration of mannitol, a hyperosmolar agent, in a rabbit model, whose vascular anatomy facilitates the use of standard interventional neuroradiology techniques and devices. To date, however, no protocols are available that enable human glioma modeling in rabbits. In this article, we report on the xenotransplantation of a human glioblastoma (GBM-1) in adult New Zealand rabbits. We induced multi-drug immunosuppression (Mycophenolate Mofetil, Dexamethasone, Tacrolimus) and stereotactically implanted GBM-1 tumor cells into rabbit brains. The rabbits were followed for 42 days, monitored by MRI and body weight measurements, and underwent postmortem histopathological analysis. On MRI, brain tumors were identified on T2-weighted scans. On histopathology, tumors were detected with hematoxylin/eosin and their human origin was confirmed with immunohistochemistry against human-specific antigens. Our method for human glioma modeling in rabbits provides the foundation to test novel treatment strategies, including intra-arterial therapeutic agent delivery.

## Introduction

Tremendous potential remains for improving outcomes in patients with brain tumors, as treatment success lags behind other non-central nervous system malignancies. This is partially due to the blood-brain barrier (BBB), which is known to be a major obstacle to effective drug delivery. Initially described in 1970 [1] and first performed in a patient in 1980 [2], intra-arterial infusion of mannitol for osmotic BBB opening has been performed in more than 4,200 procedures in over 400 patients with brain tumors [3, 4]. Despite an acceptable safety profile, this technique has not been widely accepted due to challenges with high variability and reproducibility. Additionally, the lack of simultaneous definitive confirmation of BBB opening and assessment of drug biodistribution limits this technique.

We have shown that hybrid platforms utilizing MRI and fluoroscopic x-ray based angiography techniques enhance the precision of intra-arterial delivery far better than using either modality alone. Our work demonstrated that predictable, precise, and highly selective opening of the BBB can be achieved in a rabbit model utilizing MRI in conjunction with conventional angiographic procedures [5]. In addition, the intra-arterial approach enables the immediate delivery of local, targeted chemotherapy after confirmation of BBB opening as well as monitoring the biodistribution in a label-free fashion by chemical exchange saturation transfer (CEST) MRI[6–8]. We have provided the technical foundation to be able to test various agents delivered intra-arterially targeted against brain tumors; however, except for a model of metastatic VX2 rhabdomyosarcoma [9], no brain tumor model exists in rabbits. Thus, our major motivation for the current study was to create the first human glioma model in a rabbit using the GBM1 neurosphere line, which was useful in our previous studies [10].

## Materials and Methods

### Maintenance and preparation for the transplantation of the GBM1 cell line

The HSR-GBM1 neurosphere cell line was established from a human primary glioblastoma (GBM). HSR-GBM1 cells were maintained and cultured as neurospheres at 37°C and 5% CO_2_, as previously described[10]. Prior to transplantation, cells were harvested, mechanically dissociated to single cells or small spheres, suspended in PBS at a density of 10^5^/μl, and placed on ice.

### Transplantation of GBM-1

All animal procedures were approved by the Johns Hopkins University Animal Care and Use Committee (ACUC). The procedure was carried out under general anesthesia (2% isoflurane) in adult New Zealand rabbits (n = 3). The rabbit’s head was immobilized in a stereotactic apparatus, the skin incision was performed at the midline, and the burr hole was placed over the right striatum. Then, the micro needle (gauge 26) was introduced to the brain and 10^6^ GBM1 cells were injected at the coordinates (AP = 0.0; ML = 4.5; DV = 8). The needle was withdrawn one minute after the completion of the injection, the skin was closed with sutures, and, after recovery from anesthesia, rabbits were returned to the home cage. Postoperative analgesia (ketoprofen 5mg/kg) had been provided over 48 hours to alleviate postoperative pain.

### Immunosuppressive regimen

Dex (dexamethasone sodium phosphate; 5mg/kg), Tac (tacrolimus, 0.15ml/kg), and MMF (mycophenolate mofetil, 40mg/kg; Sigma-Aldrich) were administered subcutaneously. The drug administration started on day 0 at the time of intracranial implantation of GBM1 cells. The animals were monitored daily, and any side effects from the immunosuppressive treatment were monitored by observation of the animal’s general appearance and by recording the body weight. The maximum acceptable threshold for loss of body weight was set at 30%. At the 21^st^ day after implantation, when one of the rabbits reached that threshold, the immunosuppressive regimen was reduced by phasing out the Dex, and Tac and MMF were administered at half the doses.

Physical appearance, behavior, and general and local clinical signs of the animals were observed throughout the experiments. No deviation from normality was recorded, feeding and drinking was normal not requiring supportive care.

### MRI

Imaging experiments were performed on a 4.7T animal MRI system (Bruker Biospin), with a 70-mm body coil for radiofrequency transmission and for signal reception. Coronal T2w images were acquired using the following parameters: repetition time (TR) = 3 s; echo time (TE) = 64 ms; 5 slices; thickness = 1.5 mm; field of view (FOV) = 42/32 × 32 mm2; matrix = 256/192 × 192; number of averages (NA) = 2. T1w images (repetition time = 700 ms; echo time = 10 ms; NA = 10) with and without Gd were acquired with the same geometry and location as the T2w images. Tumor volume and morphological features were assessed by ex vivo magnetic resonance imaging (MRI) at 4.7T.

### Post-mortem assessment

All animals were anesthetized and transcardially perfused with 4% paraformaldehyde (PFA). The brains were removed, post-fixed in 4% PFA overnight, and cryoprotected in 30% sucrose. Brains were cryosectioned at -20 degrees with a cryostat. Hematoxylin and eosin (H&E) staining was performed for histology. Confirmation of the tumor placement and species specification was performed with antibodies against human-specific antigens, such as Human Nuclear Antigen and STEM121. We also analyzed the status of microglia activation and T lymphocyte infiltration using Iba1 and CD3, respectively. Astrocytic specification was assessed with immunofluorescence for the expression of GFAP.

## Results

### In vitro characterization of GBM1 cells

**Fig 1:**
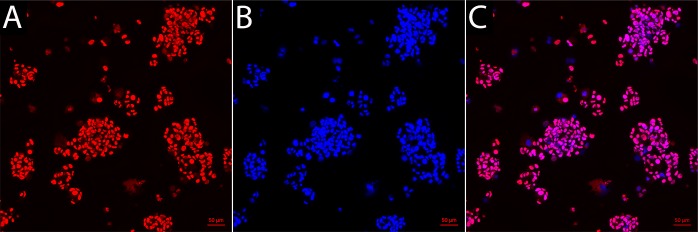
Human nuclear antigen immunofluorescence staining of the GBM1 cell line (A) with nuclear DAPI counterstain (**B)** and the overlay **(C)**.

GBM1 cells were seeded as single cells in non-adherent plates, the cells were highly proliferative and formed neurospheres. The human origin of these cells was confirmed with immunohistochemistry against human-specific nuclear antigen (HuNu), with all cells found to be immunoreactive with HuNu and with nuclei heterogeneous in size and shape **(Fig 1**).

### Overall tolerance for the immunosuppressive treatment

**Fig 2:**
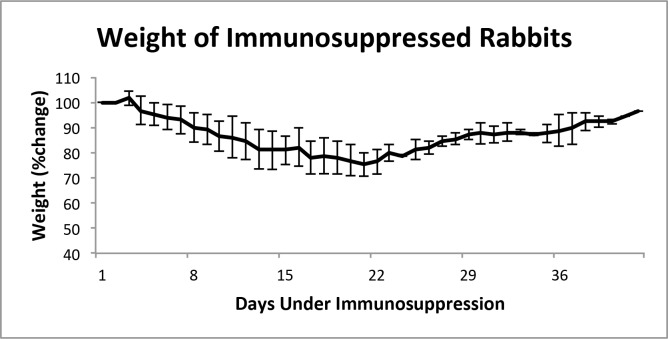
Body weight measurements over the course of immunosuppression.

Throughout the seven weeks of the experiment, animals were monitored, with daily body weight measurements obtained. Weight loss was observed very early, just after three days following the initiation of the immunosuppressive treatment (**Fig 2**). Although animals appeared to be in good health and feeding normally, and with no evidence of dehydration, weight loss continued and reached a 20% reduction after 20 days of treatment. After modification of the immunosuppressive strategy by the discontinuation of Dex and reducing the dose of Tac and MMF, rabbits regained the majority of the lost weight.

### MRI

**Fig 3:**
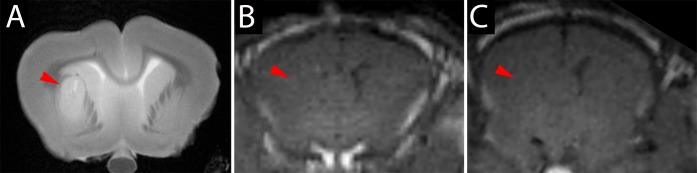
MRI of GBM-1-implanted rabbits. (A) T2-weighted scan showing hyperintense tumor unilaterally near the thalamus (red arrowhead). T1-weighted scan before (B) and after gadolinium injection (C) showing a lack of enhancement within the tumor.

Tumors were visible on T2-weighted scans as oval-shaped regions near the thalamus that showed hyper-/isointensity (**Fig 3A**). A moderate mass effect was evidenced by compression of the ipsilateral lateral ventricle. Gadolinium-enhanced T1 MRI did not show any enhancement within the tumor suggesting an intact BBB (**Fig 3B and 3C**).

### Immunohistochemical characterization

**Fig 4:**
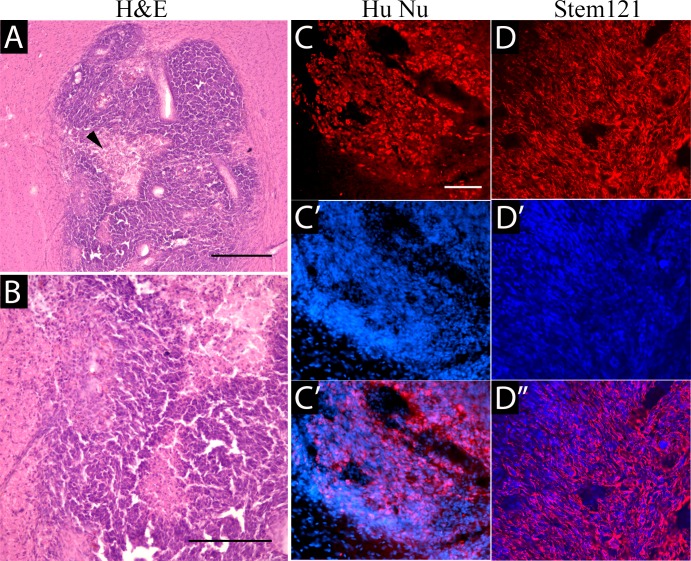
Identification of implanted tumor with histology. H&E staining (A,B) shows a cellular astrocytic tumor with large necrotic central regions (red arrowhead). Scale bars 50 μm in A,C and 100μm in B.

**Fig 5:**
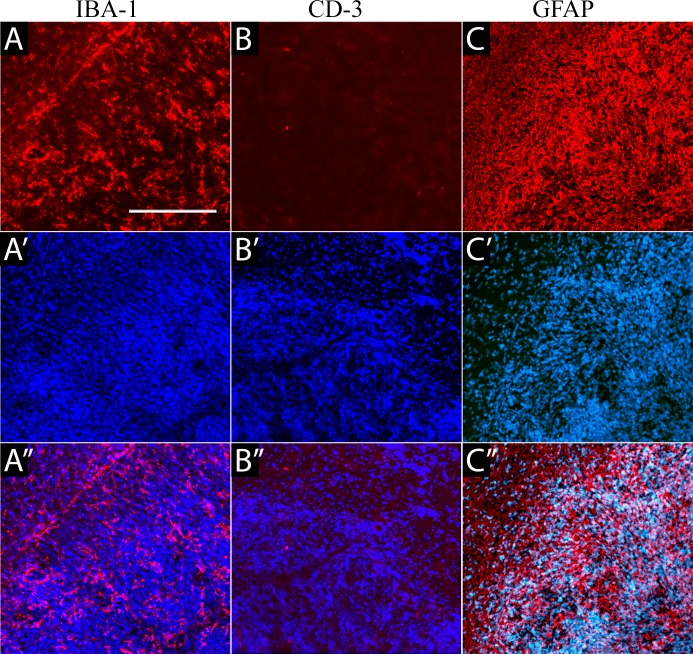
Immunohistochemical characterization of the tumor microenvironment. (A) Staining against IBA-1 detected microglial activation throughout the tumor. (B) Immunohistochemistry against the T cell marker, CD3, detected very low infiltration, with only single cells observed inside or at the periphery of the tumor. (C) GFAP immunoreactivity was detected in the tumor with all cells expressing high levels of this protein, as well as in the area around the tumor, consistent with activation of surrounding endogenous astrocytes.

Implanted human GBM1 tumors were characterized by expansive and infiltrative growth, as detected on H&E staining (**Fig 4A and 4B**) or with the human-specific markers, HuNu (**Fig 4C**) and the Stem121 (**Fig 4D**) antibody. The cytoplasmic Stem 121 marker allows a better appreciation of the infiltrative nature of this tumor. Importantly, GBM1 tumors in the rabbit brain have similarities in growth and pathology to aggressive brain tumors found in patients, such as large central necrotic areas (**Fig 4A**, arrowhead) and highly aggressive and invasive growth, with cells infiltrating into the surrounding brain. Activated microglia were noted throughout the tumor (**Fig 5A**). Immunostaining against CD3 detected very few T cells (**Fig 5B**). The glial marker GFAP was expressed at high levels in the cytoplasm of all tumor cells (**Fig 5C**).

## Discussion

In our study, we decided to implement a more aggressive immunosuppressive regimen with a combination of Dex, Tac, and MMF, and this approach appears to be effective in complete prevention of GBM-1 xenograft rejection, with good overall tolerance by the treated rabbits. BBB disruption within tumors was not detected, and can be considered a desirable model for tumors characterized initially by an intact BBB, including diffuse progressive pontine gliomas [11].

Rabbits,are relatively large with a vasculature size and anatomy suitable for navigation of intra-arterial catheters as far as the posterior circulation of the brain[5]. The difficulty with using rabbits for modeling brain tumors is the lack of immunodeficient models. Notably, to the best of our knowledge, a primary brain tumor model in rabbits has not yet been established.

One reproducible rabbit tumor model was developed in 1930–40 by Rous et al. [12, 13], which is a virus-induced leporine, anaplastic, squamous cell carcinoma—VX2. This line has been used as a model for malignancies in several organs, including the lungs[14], liver[15], kidney[16], brain[17], and spinal cord[18]. The VX2 line is quite cumbersome to use, as a culture protocol has not been established and the cells have to be propagated as muscle implants into syngeneic (New Zealand White) rabbits. Tumors are then dissected and implanted into syngeneic recipient target organs [19]. VX2 is characterized by very rapid, aggressive growth and hypervascularity, but with a high rate of spontaneous necrosis of tumor cells [19]. Although it has been suggested that there are similarities between glioma and VX2, the clinical relevance of VX2 as a primary brain tumor model is limited, and should be rather considered as a model of brain metastatic tumor.

Modeling human brain tumors is highly desirable, because it would represent a more relevant system that would enable studies on the interactions between the tumor and the microenvironment, facilitate innovative treatment approaches, as well as permit the study of human tumors xenografted in animals. However, xenotransplantation of human tumor cells into the brain of immunocompetent animals requires pharmacological immunosuppression. This approach has been used successfully to induce gliomas in pigs[20]. Pigs were immunosuppressed with a relatively mild regimen consisting of cyclosporine, 15mg/kg, and the brain tumors that developed after injection of human glioma cells were visualized by MRI; however, the blood brain barrier in these tumors was highly permeable, as evidenced by prominent enhancement on gadolinium-enhanced T1 MRI. This high permeability was likely due in part to incomplete suppression of the rejection process. However, the pig intracerebral circulation does not closely model humans due to the pig rete mirabile.

In conclusion, the GBM1 rabbit model of brain tumors is feasible, as verified by MRI and pathologic findings, and may be a suitable platform for further studies, including the intra-arterial targeted delivery of chemotherapeutics.
